# Genomic Identification, Evolution, and Expression Analysis of Collagen Genes Family in Water Buffalo during Lactation

**DOI:** 10.3390/genes11050515

**Published:** 2020-05-06

**Authors:** Xingrong Lu, Anqin Duan, Shasha Liang, Xiaoya Ma, Tingxian Deng

**Affiliations:** Guangxi Provincial Key Laboratory of Buffalo Genetics, Breeding and Reproduction Technology, Buffalo Research Institute, Chinese Academy of Agricultural Sciences, Nanning 530001, China; luxingrong074@163.com (X.L.); duanaq321@163.com (A.D.); cangshucangshu@126.com (S.L.); maxiaoya8899@163.com (X.M.)

**Keywords:** buffalo, collagen family, evolutionary relationship, gene expression

## Abstract

Collagens, as extracellular matrix proteins, support cells for structural integrity and contribute to support mammary basic structure and development. This study aims to perform the genomic identification, evolution, and expression analyses of the collagen gene family in water buffalo (*Bubalus bubalis*) during lactation. A total of 128 buffalo collagen protein sequences were deduced from the 45 collagen genes identified in silico from buffalo genome, which classified into six groups based on their phylogenetic relationships, conserved motifs, and gene structure analyses. The identified collagen sequences were unequally distributed on 16 chromosomes. The tandem duplicated genes were found within three chromosomes, while only one segmental event occurred between Chr3 and Chr8. Collinearity analysis revealed that a total of 36 collagen gene pairs were orthologous between buffalo and cattle genomes despite having different chromosome numbers. Comparative transcription analyses revealed that a total of 23 orthologous collagen genes were detected in the milk samples at different lactation periods between the two species. Notably, the duplicated gene pair of *COL4A1-COL4A2* during lactation had a higher mRNA expression level than that of cattle, while a higher expression level of *COL6A1-COL6A2* pair was found in cattle compared with that of buffalo. The present study provides useful information for investigating the potential functions of the collagen family in buffalo during lactation and helps in the functional characterization of collagen genes in additional research.

## 1. Introduction

Collagens represent the most abundant protein of the extracellular matrix (ECM) in animals. To date, the knowledge about the molecular structure, biosynthesis, and function of the collagen family has emerged [1]. Collagens comprised 28 members in vertebrates, and are multidomain proteins that commonly possessed at least one triple-helical domain. Types I to IV of the collagen family are the most common, each serving different functions with the appropriate structure. For example, collagen IV is an important component of the ECM in the mammary glands [2,3]. Chen et al. [4] found the *COL4A1* was significantly down regulated in the inflammation-associated fibroblasts extracted from bovine mammary glands with clinical mastitis compared with normal fibroblasts from a slaughtered dairy cow, implying that it might involve the ECM remodeling or immune response. Crisà et al. [5] reported that *COL4A1* was higher expressed in mature milk than colostrum milk in goats. Undoubtedly, as the major component of ECM, collagens play a vital role in mammary gland development and lactation. It is well known that the mammary gland is a dynamic organ of mammals that produce milk to meet nourishment requirements for the offspring [6,7]. Therefore, it is of great importance to reveal the putative function of the collagen family affecting the mammary gland development and lactation. Although the expression profiles and functions of collagens have been already reported, expression patterns and putative functions of the collagen gene family in water buffalo (*Bubalus bubalis*) during lactation are poorly characterized.

Water buffalo is the second largest source of milk production mainly distributed in tropical and subtropical areas, providing more than 5% of the world’s milk supply [8]. Remarkably, buffalo milk is more abundant in fat and protein than cow’s milk [9], which has attracted widespread attention from the dairy industry. In the past several decades, numerous high-throughput data were generated and utilized for identifying the candidate genes related to traits of interest in buffalo, such as the potential genes related to milk or productive traits [10,11], transcriptome profiles of buffalo embryos with normal and retarded growth [12], and maternally expressed proteins in buffalo oocyte [13]. All these data, along with the complete buffalo genome sequence [14], provide the possibility to perform the gene family analysis at a genome-wide level. In this regard, we identified the collagen family in the present study from the buffalo genome, and analyzed their evolutionary relationship, sequence features, chromosomal location, gene duplication, and dynamic expression patterns in response to different lactations (early lactation, mid-lactation, and late lactation) in milk. Our results provide some insights into the understanding of the buffalo collagen family affecting mammary gland development and lactation, and present vital evidence for future functional studies.

## 2. Materials and Methods 

### 2.1. Identification of the Buffalo Collagen Genes

Whole-genome data of six representative mammals including the human (GRCh38.p12), cattle (ARS-UCD1.2), buffalo (UOA_WB_1), goat (ARS1), sheep (Oar_rambouillet_v1.0), and horse (EquCab3.0) were downloaded from the National Center for Biotechnology Information (NCBI) Genome database [15], aiming to identify the buffalo collagen genes. The HMM profile of the collagen domain (PF01391) from the Pfam database [16] was used to search the buffalo dataset using the HMMER [17,18] software with an E-value cut-off of 1.0 × e^−5^. The identified buffalo collagen protein sequences were also validated by the BLAST with collagens from the other five mammals as queries. Further, the ClustalW algorithm was used for the multiple sequence alignment of collagen with the full-length protein sequences. The aligned sequences were used for constructing the neighbor-joining (NJ) phylogenetic tree of the collagen family using the MEGA7 [19] software with the Poisson model, pairwise deletion, and 1000 bootstrap resampling.

### 2.2. Sequence Analysis

The ExPASy proteomics server was utilized for the prediction of theoretical molecular weight (MW) and isoelectric points (pI) of the buffalo collagen family [20]. The gene structure was measured and visualized using the TBtools version 0.6657 [21]. Exon–intron structure analysis was conducted by the buffalo genome annotation file using the in-house scripts. We analyzed conserved protein motifs of collagen proteins in buffalo, using the MEME programs [22] at a maximum number of motifs, 10. 

Chromosome locations of each collagen gene were obtained from their genome resources. Collagen duplication events were identified using the Multiple Collinearity Scan Toolkit (MCScanX) previously described by Wang et al. [23]. Overall, a total of 58,532 buffalo protein sequences were analyzed using the BLAST search with E-value < 1.0 × e^−5^ and then the corresponding gene positions files were obtained from the buffalo genome annotation file with the GFF format, which can be set as the input files of MCScanX program. 

For the duplicated collagen genes, we further performed the divergence estimates and diversity analysis. In brief, MEGA7 software with the MUSCLE algorithm was first used for the pairwise alignment of the homologous collagen gene pairs. Subsequently, the DnaSP v6.0 [24] software was employed to calculate the pairwise synonymous (Ks) and nonsynonymous (Ka) numbers of substitutions corrected for multiple hits.

### 2.3. Comparative Transcriptomic Analyses for the Milk Samples of Buffalo and Cattle

To explore the expression difference of collagens between buffalo and cattle in milk samples at different lactation, including early lactation, mid-lactation, and late lactation, two published RNA-seq data (BioProject: PRJNA419906 and PRJNA453843) were selected and employed further analyses. Briefly, the FASTQC [25] program was utilized to remove the adapter sequence from the raw sequence reads. We aligned the clean data from buffalo and those from cattle to their genomes (buffalo: UOA_WB_1 and cattle: ARS-UCD1.2) using HISAT2 [26] with default parameters, respectively. The StringTie [27] was utilized for calculating the gene or transcript count matrix. Transcripts per million (TPM) values for each gene were obtained using the DESeq2 [28] R-package. The one-to-one orthologous collagens in milk samples were selected and then merged, as described by Yu et al. [29]. Clustering and generation of a heat map of TPM values for the selected genes were performed using the pheatmap package in R.

### 2.4. Real-time Quantitative PCR 

Murrah buffalo was used as the source of animal material in the present study, which was kept at the Buffalo Research Institute, Chinese Academy of Agricultural Sciences (BRI-CAAS). Biopsy samples of mammary gland tissue from eight buffaloes were collected on days 7 (D7), 140 (D140), and 280 (D280) after calving, which was further used for the real-time quantitative PCR (qRT-PCR). The biopsy procedure was performed based on the method reported by Schmitz et al. [30]. All fresh samples were immediately preserved in liquid nitrogen until use.

Total RNA for different buffalo tissues was isolated using the RNA Plus reagent (Tiangen, China). Quality of the total RNA was assessed using the NanoDrop2000 (Thermo Fisher Scientific, Wilmington, DE, USA) and gel electrophoresis. For 2 μg total RNA, the first-strand cDNA was synthesized using a reverse transcriptase kit (Takara, China). SYBR Premix Ex Taq (Takara) were used for qRT-PCR analysis, which was monitored on the LightCycler 480 (Roche, Switzerland), and each reaction was performed in triplicate. The expression levels of the buffalo collagen family were analyzed by the 2^−ΔΔCt^ method [31] and normalized using the expression of RPS9 and RPS15 analysis. Primers used for the qRT-PCR analysis are shown in Table S1. 

## 3. Results

### 3.1. Genomic Identification of Buffalo Collagen Genes 

**Figure 1:**
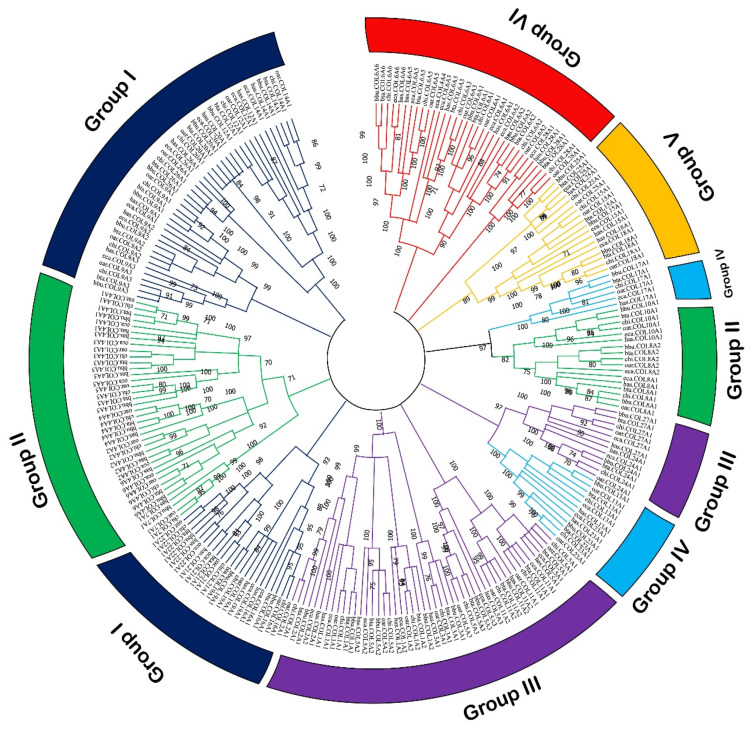
Phylogenetic relationship of collagen proteins in six mammals. Line with different colors indicates different groups. Circle of different color also indicates different groups. Buffalo: bbu; Cattle: bta; Goat: chi; Sheep: oar; Horse: eca; Human: has.

To identify the collagen family members, a total of 128 non-redundant protein sequences encoded by 45 collagen genes were predicted from the buffalo whole genome using the BLAST and HMMER software (Table S2). The open reading frames (ORFs) of the collagen protein isoforms ranged from 1317 to 9657 bp in length encoding the protein of 438 to 3218 residues, with the predicted MW from 44.65 to 347.21 kDa. The pI values of these isoforms ranged from 4.48 to 10.51. Furthermore, the phylogenetic analysis revealed that all 45 collagen genes could be divided into six groups (Figure 1). Group I was the top one with the larger numbers of collagen genes (*n* = 9), while the group VI was the smaller one (*n* = 2). The constructed dendrogram further showed that the buffalo collagen family was usually the most closely evolutionary relationship with the other five representative mammals.

### 3.2. Sequence Analysis of Buffalo Collagen Genes

**Figure 2:**
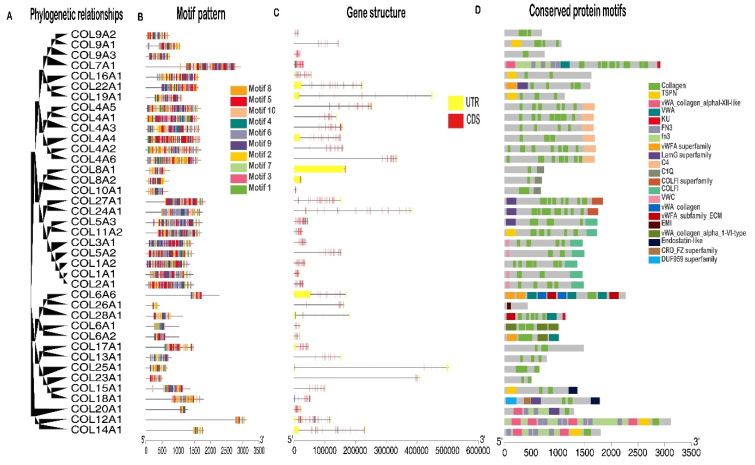
Phylogenetic relationships, motif pattern, gene structure, and conserved protein motifs of buffalo collagen gene family. (**A**) Phylogenetic tree of 45 collagen proteins. (**B**) Motif pattern of buffalo collagen gene family. Ten putative motifs are indicated in different colored boxes. For details of motifs refer to Table 1. (**C**) UTR/CDS organization of collagen genes. Yellow box, black line, and red box represent untranslated region (UTR), intron and coding sequencing (CDS), respectively. (**D**) Distributions of conserved protein motifs in collagen genes.

To explore the structural characteristics of buffalo collagens, the motifs pattern, gene structures, and conserved domains were performed taking into account their phylogenetic relationships (Figure 2). As showed in Figure 2B, a total of 10 conserved motifs were identified in the identified collagen genes. After the Pfam search, motifs 3 and 5, which both composited 41 amino acids, were annotated as the collagen domain (Table 1). The results were also supported by the identified collagens blasted against the conserved domain database (CDD) from NCBI (Figure 2D). Interestingly, the fibrillar collagen C-terminal domain (COLFI), fibronectin type 3 domain (FN3), C-terminal tandem repeated domain in type 4 procollagen (C4), von Willebrand factor (vWF) type A domain (VWA), von Willebrand factor type C domain (VWC), and EMI domain also was determined in some collagen genes. Moreover, although the introns and UTRs structure varied greatly, gene structural analysis indicated that buffalo collagen genes in the same groups had similar numbers of exon and intron (Figure 2C), suggesting that different collagen groups had different patterns of intron numbers, which verify our previous classification process.

### 3.3. Chromosomal Distribution and Collinearity Analysis of Collagen Genes

**Figure 3:**
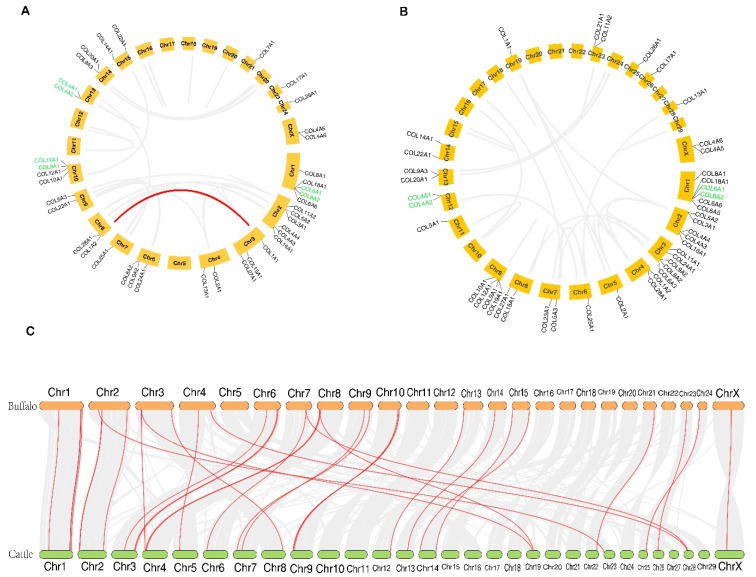
Gene duplication of buffalo (**A**) and cattle (**B**) collagen genes, as well as their collinear analysis (**C**). The tandem duplicated genes were marked by the green color, and segmentally duplicated genes are indicated by the red line.

All identified buffalo collagen genes were randomly distributed on 16 chromosomes (Figure 3A), while the cattle collagens were randomly located on 19 chromosomes (Figure 3B). The majority of buffalo collagen genes were mainly located on the proximate or the distal ends of the chromosomes. 

To investigate the evolutionary progress of the collagen family, we analyzed the duplication events of buffalo and cattle (Figure 3A and B). Among these collagen genes, three pairs of genes, including *COL4A1-COL4A2*, *COL6A1-COL6A2,* and *COL9A1-COL19A1* exhibited the tandem duplication in buffalo. Two pairs of genes (*COL4A1-COL4A2* and *COL6A1-COL6A2*) were seen as tandem duplication genes in cattle. Although only one segmental duplication event (*COL1A1* and *COL1A2*) was discovered in the buffalo collagen family, the scenario was not published in cattle. Positive selection analyses further showed that a total of four duplicated collagen gene pairs in buffalo with the number of nonsynonymous substitutions per nonsynonymous site (Ka)/ the number of synonymous substitutions per synonymous site (Ks) ratios < 1 were identified, three of which had Ka/Ks ratios less than 0.5 (Table 2).

Collinearity analysis between buffalo and cattle genomes showed that a total of 34,381 orthologous genes were determined, which account for 82.21% of the total genes (Figure 3C). Interestingly, a larger chromosome homologous existed between river buffalo (2*n* = 50) and cattle (2*n* = 60) despite the fact that they have different chromosome numbers. Among the collagen family genes, a total of 36 pairs of collagen genes were orthologous between the two species, and their information was listed in Table S3. 

### 3.4. Comparative Transcriptomic Analyses of Orthologous Collagens between Buffalo and Cattle

**Figure 4:**
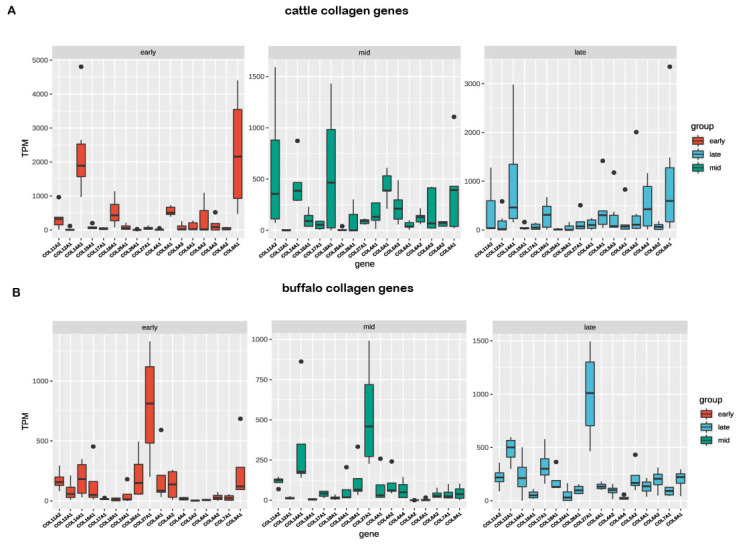
Collagen expression analysis of cattle (**A**) and buffalo (**B**) in milk at different lactations. Red box indicates early lactation; green box indicates mid-lactation; light blue box indicates late lactation.

**Figure 5:**
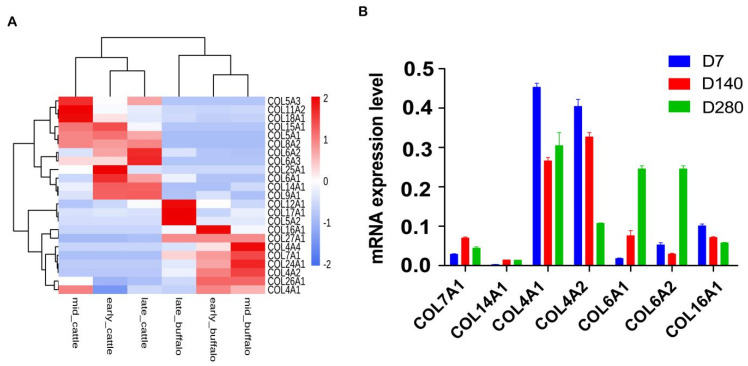
Heat map (**A**) of orthologous collagen genes of cattle and buffalo and validation of selected buffalo collagens by qRT-PCR (**B**). Distances, representing the relative similarity among genes and tissues, were calculated using Pearson’s correlation coefficients. Color represents the TPM (transcripts per million) values of gene expression after scaling and centering. D7 indicates early lactation, D140 indicates mid-lactation, and D280 indicates late lactation.

Using the RNA-seq data, we further dissect the expression difference of orthologous collagens between buffalo and cattle in milk samples at three lactation points (early, mid, and late lactation). The results showed that a total of 29 collagen genes were found in the milk samples of cattle (Figure 4A) and buffalo (Figure 4B), accounting for 64.44% and 69.05% of the total, respectively. We further found that a total of 23 orthologous collagen genes were detected in milk samples between the two species (Figure 5A). The expression profiles of orthologous collagen genes were more similar in the same species than the expression patterns for the same tissue in different species. The different expression trend of orthologous collagens was also found within the same species. Interestingly, the expression level of the duplicated *COL4A1-COL4A2* genes was higher in buffalo than that of cattle during lactation, while the higher expression level of the *COL6A1-COL6A2* pair was found in cattle compared with that of buffalo. Moreover, our results of qPCR analysis showed that the selected collagen genes in buffalo had similar mRNA expression tendency with that of RNA-seq data (Figure 5B).

## 4. Discussion

Collagens, as ECM molecules, support cells for structural integrity and a variety of other functions [32], thus contributing to the support of mammary basic structure and development [33]. Currently, our understanding of the functional role of the buffalo collagen family is limited. In the present study, we identified 128 collagen proteins sequences in buffalo based on its complete genome sequence. The identified collagen protein sequences corresponded to 45 collagen genes in buffalo, which were classified into six groups based on their evolutionary relationships. The result was in line with the previous classification of collagens described by Ricardblum [34]. The conserved motif and gene structure analyses of buffalo collagen genes also supported this classification perspective. Conserved motif analysis indicated that all the identified collagens harbored at least the collagen domain, which is also supported by the previous studies [35,36,37]. Interestingly, the collagen group I genes contained a fibrillar collagen C-terminal domain (*COLFI*). For the gene structure analysis, most of the collagen genes contained 40 to 60 exons. The intron number analysis revealed that the majority of collagen genes contained more than forty introns, whereas only the *COL8A1*, *COL8A2*, and *COL10A1* contained fewer than three introns. These results are consistent with the exon–intron structure of collagen genes from other representative mammals, suggesting that the collagen family had a conserved gene structure.

Four events during genetic evolution including chromosome doubling, chromosome fragment insertion mutation, tandem duplication, and transposition provide a possibility for the novel gene function acquisition [38,39]. Gene duplication including tandem duplication and segmental duplication can mainly help to accelerate the gene family expansion and genome evolutionary mechanisms [40,41,42]. In buffalo, all identified collagens were unevenly distributed on 16 chromosomes. Here, a total of three buffalo collagen gene pairs were confirmed to be tandem duplicated genes, but only one segmental duplication event was confirmed to be discovered, which revealed that tandem duplication had a predominant role in the expansion of buffalo collagen family. This finding was supported by Liu et al. [39], who highlighted that segmental duplications in most mammalian lineage are organized in a tandem configuration. For the duplicated collagen pairs, interestingly, we found that four pairs of duplicated collagens with the Ka/Ks ratios < 1 were identified; three of them with the Ka/Ks ratios were less than 0.5, which might experience strong purifying selection pressure.

Previously studies showed that the members of the collagen family are the most abundant proteins in ECM that are tightly regulated throughout the development of the mammary gland [34,42,43]. Therefore, to explore the expression pattern of collagen genes between buffalo and cattle in milk samples at different lactation points is necessary, which contribute to dissect the potential roles of these genes in the milk morphogenesis. In the present study, collinearity analysis revealed that a large number of homologous chromosomal regions were observed between buffalo and cattle. A similar result was also noted in other studies [8,44]. We observed that a total of 36 pairs of collagen genes were orthologous between the two species. For them, a total of 23 orthologous collagens were detected in the milk samples of buffalo and cattle. Notably, they had a more similar expression level in the same species than that of different species, reflecting the hereditary discrepancy between buffalo and cattle concerning mammary gland development and lactation, respectively. Moreover, the different expression trend of orthologous collagens was also found within the same species during lactation, suggesting that these genes had a spatiotemporal expression. Interestingly, the duplicated gene pair of *COL4A1-COL4A2* during lactation was at a higher expressed level than that of cattle, whereas a higher expression level of *COL6A1-COL6A2* pair was found in cattle compared with that of buffalo. Compared with that of buffalo, moreover, a total of 12 collagen genes in cattle had a higher expression level. For them, three (*COL5A3*, *COL11A2*, and *COL18A1*) collagen genes were upregulated in mid-lactation. Two (*COL6A2* and *COL6A3*) collagens were upregulated in late lactation, and two (*COL25A1* and *COL6A1*) were upregulated in early lactation. Dai et al. [45] found that *COL8A1* and *COL1A2* both were upregulated in the bovine mammary gland during lactation compared to the dry period. The reason for the difference could be the limitation of available expression data for cattle collagen and the comparison method. Moreover, a total of 11 buffalo collagens had a higher expression level compared to that of cattle, three (*COL12A1*, *COL17A1*, and *COL5A2*) of which were upregulated in late lactation, as well as *COL16A1* and *COL4A4,* which were respectively upregulated in early and mid-lactation. These results were also supported by the qRT-PCR. Our findings suggested that these collagen genes displayed the specific biology function at different lactation stages. However, these findings have yet to be confirmed.

## 5. Conclusions

In this work, we performed genomic identification of the buffalo collagen family, with 128 collagen protein sequences confirmed. Next, we found that collagen sequences in buffalo were unequally broadcast on 16 chromosomes. Three tandems and one segmental duplicated gene pair were determined in buffalo, and 36 pairs of collagen genes were orthologous between buffalo and cattle genomes. Moreover, comparative transcription analyses revealed that the highly expressed orthologous collagen genes were different between the buffalo and cattle in the milk samples. The study provides valuable information on the collagen gene family in buffalo affecting mammary gland development and lactation and will assist in determining the collagen gene functions.

## Figures and Tables

**Table 1:** Ten different motifs commonly observed in buffalo collagen family.

Motif	Protein Sequence	Length	Pfam Domain
MEME-1	GLPGLKGEKGEAGLPGFKGEKGVKGEKGE	29	-
MEME-2	KGEDGLPGLPGEKGEKGEKGDPGPPGPPG	29	-
MEME-3	GEKGERGLPGLPGKKGAKGEPGIPGAKGEKGPPGPPGPPGE	41	Collagen
MEME-4	GPPGPPGPPGPPGPPGLPGPPGPPGLPGPP	30	-
MEME-5	PGPPGPKGPRGEKGDPGSTGPPGEPGLPGLQGPPGEKGDKG	41	Collagen
MEME-6	GPKGERGPKGQKGEKGQPGEP	21	-
MEME-7	TGPPGPIGLPGLPGPKGEKGE	21	-
MEME-8	GEPGJPGEKGEPGLPGPPGLPGEKGPKGK	29	-
MEME-9	GEQGERGPKGEKGEA	15	-
MEME-10	RGEPGLPGPPGPPGP	15	-

**Table 2:** Analysis of the Ka/Ks ratios for each pair of duplicated collagen genes in buffalo.

Gene Pairs	CHR	Ka	Ks	Ka/Ks Ratio
*COL1A1/COL1A2*	3/8	0.2872	1.2576	0.2283
*COL4A2/ COL4A1*	13	0.5336	1.9459	0.2742
*COL6A1/ COL6A2*	1	0.7068	1.0659	0.6631
*COL9A1/ COL19A1*	10	0.6218	2.6177	0.2375

CHR: Chromosome of buffalo; Ka: the number of nonsynonymous substitutions per nonsynonymous site; Ks: the number of synonymous substitutions per synonymous site.

## Supplementary Materials

The following are available online at https://www.mdpi.com/2073-4425/11/5/515/s1: Table S1, Primers of qRT-PCR for some collagen genes in buffalo. Table S2, Features of the predicted collagens protein sequences in buffalo. Table S3, Orthologous collagen gene pairs of buffalo and cattle.
